# Labor induction with double balloon catheter, oral misoprostol or both: a randomized controlled trial

**DOI:** 10.1007/s00404-026-08526-9

**Published:** 2026-08-12

**Authors:** Inna Bleicher, Dafna Ben Yehuda, Dmitry Basov, Mayson Einas Khniefes, Sharon Einav, Shlomi Sagi, Amjad Shulhut, Samira Talhamy, Rami Sammour

**Affiliations:** 1 The Ruth and Bruce Rappaport Faculty of Medicine, Technion - Israel Institute of Technology, Haifa, Israel; 2 Department of Obstetrics and Gynecology, Bnai -Zion Medical Center, Haifa, Israel; 3 Faculty of Medicine, Hebrew University of Jerusalem, Surgical Intensive Care Unit, Shaare Zedek Medical Center, Jerusalem, Israel; 4 Hod HaSharon Maccabi Healthcare Services, Hod HaSharon, Israel

**Keywords:** Labor induction, Misoprostol, Double balloon, Cervical ripening, Cytotec

## Abstract

**Objective:**

To compare cesarean delivery rates and induction outcomes among women undergoing labor induction with a cervical ripening double balloon (DB), oral misoprostol (OM), or a combination of both (DOM).

**Methods:**

This single-center randomized controlled trial included women with singleton term pregnancies, cephalic presentation, Bishop score ≤ 4, and intact membranes. Participants were randomized to DB for 6 h, oral misoprostol, or combined treatment. The primary outcome was cesarean delivery rate. Secondary outcomes included Bishop score after 6 h, time from induction to delivery, failed induction, and maternal and neonatal outcomes. Due to slow recruitment, the study was stopped early, and complete-case analysis was performed.

**Results:**

A total of 216 participants were included in the analysis (DB n = 65, OM n = 75, DOM n = 76). Cesarean delivery rates were 20%, 11%, and 20% in the DB, OM, and DOM groups, respectively (p = 0.2). The combined treatment group demonstrated a significantly shorter induction-to-delivery interval (median 14.7 h vs 20.7 in DB [p = 0.006] and 22.9 h in OM [p < 0.001]); higher 6-h Bishop scores (median 6 vs 5 in DB [p = 0.012] and 4 in OM [p < 0.001]);and lower rates of failed induction (2.6% vs 18% in DB [p = 0.010] and 5.4% in OM [p = 0.4]). Maternal and neonatal outcomes were similar across groups.

**Conclusion:**

Despite limited power this study suggests that combined use of cervical ripening balloon and oral misoprostol shortens induction duration and improves early cervical ripening without increasing cesarean delivery rates. This approach may enhance the efficiency of labor induction.

**Clinical trial regirstration:**

Date of registration: 03–03–2019. Date of initial participant enrollment, July 1st, 2019. Clinical trial identification number #NCT03866772. URL of the registration site: https://clinicaltrials.gov/study/NCT03866772#study-overview

**Supplementary Information:**

The online version contains supplementary material available at https://doi.org/10.1007/s00404-026-08526-9.

## Introduction

When labor is induced, the condition of the uterine cervix, as assessed by the Bishop score [1], influences both the time to delivery and the likelihood of achieving vaginal birth. If the cervix is unfavorable, ripening is recommended [2]. Once adequate ripening has occurred, artificial rupture of the membranes, typically when the Bishop score reaches 5 or higher, facilitates progression to the next stage of induction of labor (IOL).

Cervical ripening may be achieved by either mechanical or pharmacological methods. Mechanical methods include the Foley catheter, the double cervical ripening balloon (CRB), and osmotic cervical dilators, whereas pharmacological methods consist mainly of prostaglandins [3]. [4] Misoprostol, a synthetic prostaglandin administered either orally or vaginally, has become increasingly common for labor induction [5]. Despite the effectiveness of the vaginal route, oral administration may be associated with less uterine tachysystole at a similar dose, resulting in improved patient satisfaction and possibly better neonatal outcomes [6], and has therefore been adopted as the preferred method by many institutions, including ours. Balloon catheters, typically placed for 12 h, are as effective as oral misoprostol (OM) for IOL [5] and have also been associated with less uterine tachysystole [7]. Recently, a growing body of evidence supports removal after 6 hours [8–11], achieving similar obstetric outcomes and reducing time to delivery. The choice between mechanical or pharmacological cervical ripening depends on several factors, including safety, side effects profile, patient preference, contraindications to one of the methods, and local clinical protocols [6].

In recent years, combining two methods for induction of labor has become more popular [5]. Kehl et al. (2011) randomized patients to OM versus combined OM and CRB, showing a higher rate of vaginal delivery within 48 h with combined therapy [12].

We therefore decided to conduct a study to compare the use of cervical ripening balloon for 6 h, oral misoprostol, or the concomitant use of both (DOM), and its effect on cesarean delivery rate in patients undergoing induction of labor. We hypothesized that the combination of the two methods would lead to reduced cesarean delivery rate.

## Materials and methods

### Settings and participants

This was a single center, randomized controlled trial, aimed to assess the rate of cesarean delivery (CD) following induction of labor using 6 h double balloon catheter, oral misoprostol or concomitant use of both. The study was conducted at a tertiary university affiliated medical center. The hospital provides medical care for a heterogeneous population and serves approximately 3400 deliveries annually. All labor and delivery costs are covered by government insurance.

All patients scheduled for IOL were screened for fulfillment of eligibility criteria. We included patients with a singleton gestation, > 37 gestational weeks, cephalic presentation, Bishop score ≤ 4 and intact membranes. Patients with a maximum vertical pocket < 2 cm, growth restriction < 10th percentile, and prior uterine surgery or any contraindication for vaginal delivery were excluded.

Informed consent was received in accordance with Institutional Review Board requirements (IRB #0166–18 BNZ) once fulfillment of inclusion/exclusion criteria was verified. All data were collected in real time by the attending staff, in accordance with predefined clinical report forms (CRFs). The trial was registered at clinicaltrials.gov #NCT03866772.

The outcomes are reported according to CONSORT guidelines.

After informed consent, patients were randomized to cervical ripening using cervical double balloon catheter (DB group), oral misoprostol (OM group), or the concomitant use of both (DOM group).

The protocol for cervical ripening balloon catheter insertion is described elsewhere [11], and included removal after 6 h, in accordance with our local protocol. The protocol for cervical ripening with misoprostol included administration of 50 mcg of oral misoprostol every 4 h until the start of active labour, defined as ≥ 3 painful contractions in 10 min and cervical dilatation ≥ 3 cm, to a maximum of six doses. Electronic fetal monitoring was performed for 30 min after administration of misoprostol. The concomitant treatment included insertion of the CRB followed by immediate intake of 50 mcg of oral misoprostol. Initiation of oxytocin infusion was allowed only 4 h after the last dose of misoprostol.

### Outcomes

The primary outcome was the rate of CD. Secondary outcome measures included Bishop scores assessed 6 h after initiation of induction, time to delivery (defined as the time interval from administration of the first dose of misoprostol or insertion of the CRB until delivery), rate of failed induction (defined as no cervical change or uterine contractions observed within 24 h of initiation of any of the above methods), and other maternal and neonatal outcomes.

### Sample size calculation

The sample size was calculated for a 2-sided α = 0.05 and power of 80%, for a 2-degrees of freedom Chi-squared test comparing 3 intervention groups’ CD proportions, our primary outcome, using the local cesarean delivery rate of 19%. If at least one group showed a difference of at least 5% in CD proportion (effect size w = 0.11), the required sample size would be 797 overall (266 per group).

### Randomization, sequence generation, and allocation concealment

A random online sample generator was used to plan an allocation ratio of 1:1:1. The allocation sequence was generated by an independent research assistant not involved in patient recruitment or outcome assessment, using permuted blocks of variable sizes to ensure balance between the three groups while maintaining allocation concealment. Each generated number was printed and placed inside a sealed, opaque, and sequentially numbered envelope. The envelope was opened after determination of fulfilment of inclusion criteria and signed informed consent.

### Implementation

All co-investigators had access to the sequentially numbered and sealed envelopes. After enrollment, the co-investigator was allowed to open the envelope and assign participants to the intervention groups.

### Blinding

Due to the nature of the interventions used, neither the patient nor the researchers could be blinded to the intervention. The statistician received a file with a coded intervention name.

### Statistical analysis

We described participants’ demographics and baseline clinical characteristics by study group with median and interquartile range (IQR), frequencies and percentages and compared groups using Kruskal–Wallis rank sum test, Chi-squared test and Fisher’s exact test. Primary and secondary endpoints were similarly described. Primary endpoints were tested using Chi-squared test and Fisher’s exact test, CD proportions were described with a 95% confidence interval, calculated using the Clopper-Pearson exact method. Secondary endpoints were tested with pairwise Wilcoxon rank sum test and pairwise Fisher’s exact test, comparing each pair of induction methods. Pairwise comparisons p-values were adjusted for multiple comparisons using Benjamini and Hochberg’s method (for each endpoint but not overall). To account for the effect of nulliparity on induction outcomes, we repeated this analysis in nulliparous and parous women, and tested the interaction of nulliparity and study intervention in a logistic regression model for cesarean delivery and linear regression model for induction to delivery time. Statistical analysis was performed in R version 4.2.2. A 2-sided α = 0.05 was considered statistically significant.

## Results

Patients were recruited over a period of 50 months (July 2019–September 2023), and followed until hospital discharge. The study was discontinued before reaching the planned sample size, because of slower-than-anticipated recruitment. Resultantly, with a 2-sided α = 0.05, power of 80% and a 2-degrees of freedom Chi-squared test, and a sample size of 216 the study was powered to detect an effect size of w = 0.21, which translates approximately to a 9% or more difference between any two groups in CD proportion (in contrast to our study analysis plan to detect of 5% difference or more).

During the study period, approximately 1,500 patients requiring labor induction were screened, and 250 were eligible, provided consent, and were randomized to the intervention groups (DB n = 80, OM n = 83, DOM n = 87). In the intention-to-treat population (n = 250), protocol violations occurred in 5/250 cases (OM n = 3, DOM n = 2,), and two cases of spontaneous labors were recorded (DB n = 1, OM n = 1).

Eight women withdrew consent and were excluded (DB n = 3, OM n = 2, DOM n = 3). Primary outcome data were missing for 26 participants (DB n = 12, OM n = 6, DOM n = 8). Missing data occurred due to technical issues, and missingness was assumed to have occurred at random. Complete case analysis was therefore performed on n = 216 participants (DB n = 65, OM n = 75, DOM n = 76).

In the analytic cohort, protocol violations were observed in 3/216 cases of (all in the OM group), and one spontaneous labor occurred in the DB group (Supplementary Table S1).

### Baseline characteristics

A total of 216 patients (65, 75 and 76 in the DB, OM, and DOM groups, respectively) were included in primary outcome analysis. The three groups were comparable in all baseline characteristics (Table 1).

**Table 1:** Patient characteristics

Characteristic	DB N = 65	OM N = 75	DOM N = 76	p-value^1^
Maternal age	29 (25, 33)	31 (27, 35)	31 (28, 35)	0.1
Gestational age (weeks)	39.4 (38.4, 40.5)	39.2 (38.0, 40.2)	39.4 (38.6, 40.5)	0.2
BMI (Kg/m2)	29.3 (26.0, 31.6)	30.0 (27.0, 34.0)	29.5 (26.0, 34.1)	0.7
(Missing)	21	15	13	
Nulliparity	37 (57%)	44 (59%)	38 (50%)	0.5
Bishop score before induction	1 (0, 3)	1 (0, 3)	2 (0, 3)	0.5
(Missing)	3	5	3	
Birthweight (g)	3,403 (3,058, 3,768)	3,320 (3,024, 3,673)	3,393 (3,050, 3,678)	0.7
(Missing)	1	3	0	
Indication for labor induction				
Epidural during labor	60 (92%)	67 (89%)	71 (93%)	0.6
Post date	18 (28%)	16 (21%)	21 (28%)	0.6
Hypertensive disorders	7 (11%)	18 (24%)	8 (11%)	0.034
Oligohydramnios	3 (4.6%)	4 (5.3%)	2 (2.6%)	0.7
Non reassuring fetal heart rate	7 (11%)	5 (6.7%)	6 (7.9%)	0.7
Macrosomia	12 (18%)	9 (12%)	11 (14%)	0.6
Reduced fetal Movements	10 (15%)	10 (13%)	10 (13%)	> 0.9
Glucose abnormalities	10 (15%)	17 (23%)	13 (17%)	0.5
Maternal request	3 (4.6%)	6 (8.0%)	7 (9.2%)	0.6
Other	4 (6.2%)	6 (8.0%)	7 (9.2%)	0.8

Numbers are presented as Median (Q1, Q3) or n (%)

^1^Kruskal-Wallis rank sum test; Pearson'\’s Chi-squared test; Fisher's exact test

### Outcomes

For the primary outcome of CD, the rates were 20%, 11% and 20% in the DB, OM, and DOM groups, respectively (p = 0.2, Table 2). No differences were observed between the groups in the indications for CD (Table 2).

**Table 2:** Primary outcome

	DB N = 65	OM N = 75	DOM N = 76	p-value^1^
Cesarean delivery, n (%), (95% CI)	13 (20%) (11%-32%)	8 (11%) (5%-20%)	15 (20%) (11%-30%)	0.2
Indication for cesarean delivery, n (%)				0.3
Non-progressive labor	4/13 (31%)	4/8 (50%)	7/15 (47%)	
Non-reassuring fetal heart rate	9/13 (69%)	3/8 (38%)	5/15 (33%)	
Other	0/13 (0%)	1/8 (13%)	3/15 (20%)	

^*1*^Pearson's Chi-squared test; Fisher’s exact test, *CI* confidence interval

Regarding the secondary outcomes (Table 3), patients in the DOM group had a shorter induction to delivery interval, median 14.7 h compared to 20.7 h in the DB group (p = 0.006) and 22.9 h in the OM group (p < 0.001). The DOM also had higher post-intervention Bishop scores: 6 vs 5 in the DB group (p = 0.012) and vs 4 in the OM group (p < 0.001). The rate of failed induction was significantly lower in the DOM group compared with the DB group (2.6% vs 18%, p = 0.01), and similar to the OM group (2.6% vs 5.4%, p = 0.4).

**Table 3:** Secondary Outcomes

Characteristic	DB N = 65	OM N = 75	DOM N = 76	DB vs. OM	DB vs. DOM	OM vs. DOM
Time from IOL to delivery (hrs)				0.2	0.006	< 0.001
Median (Q1, Q3)	20.7 (15.5, 25.9)	22.9 (17.0, 31.2)	14.7 (10.3, 25.0)			
(Missing/not applicable)	9	7	4			
Time to vaginal delivery (hrs)				0.2	0.003	< 0.001
Median (Q1, Q3)	19.3 (15.4, 24.4)	21.3 (16.7, 29.3)	14.3 (9.5, 21.6)			
(Missing/not applicable)	20	14	18			
Bishop 2^2^				0.002	0.012	< 0.001
Median (Q1, Q3)	5.0 (4.0, 6.0)	4.0 (2.0, 5.0)	6.0 (5.0, 7.0)			
(Missing/not applicable)	4	10	7			
Failed induction	12 (18%)	4 (5.4%)	2 (2.6%)	0.046	0.010	0.4
(Missing)	0	1	0			
Oxytocin infusion	58 (89%)	54 (73%)	57 (75%)	0.055	0.073	0.9
(Missing)	0	1	0			
Fetal heart rate decelerations during labor	18 (30%)	19 (27%)	14 (19%)	0.8	0.5	0.5
(Missing)	4	4	1			
Tachysystole	5 (8.2%)	7 (9.7%)	8 (11%)	> 0.9	> 0.9	> 0.9
(Missing)	4	3	3			
Post-partum hemorrhage	4 (6.7%)	6 (8.6%)	6 (8.0%)	> 0.9	> 0.9	> 0.9
(Missing)	5	5	1			
Chorioamnionitis	4 (6.7%)	2 (2.9%)	5 (6.7%)	0.7	> 0.9	0.7
(Missing)	5	6	1			

Numbers are presented as Median (Q1, Q3) or n (%)

Neonatal outcomes were comparable across groups. Four neonates required NICU admission (DB n = 2, OM n = 1, DOM n = 1), and two neonates had a 5-min Apgar score < 7 (one each in the DB and DOM groups).

Only one serious adverse maternal outcome was reported, which appeared unrelated to the intervention. This patient, induced at 37 weeks for an umbilical cord varix and allocated to the OM group, underwent vaginal delivery of a 3,100 g neonate. Following spontaneous placental delivery, retained placental tissue was suspected. During manual uterine exploration, severe postpartum hemorrhage due to uterine atony developed. Despite treatment with uterotonics, uterine massage, repeated exploration, cervical assessment, and attempted tamponade with a Bakri balloon, bleeding persisted, necessitating hysterectomy. The patient subsequently developed disseminated intravascular coagulation and required transfusion of approximately 70 blood products.

In subgroup analysis in nulliparous and multiparous women, demographic and clinical characteristics were similar across study groups, in both parity subgroups (Supplementary Table S2). CD was numerically less common in multiparous women overall (5/97 = 5.6% vs 31/119 = 26%), and no statistically significant differences were seen across study groups in both parity subgroups (all p ≥ 0.1, Supplementary Table S3). Secondary outcomes by parity are reported in Supplementary Table S4. The interactions of parity and study group in regression models of CD and time from induction to delivery were both not statistically significant (Tables S5, S6).

## Discussion

In this randomized controlled trial, cesarean delivery rates did not differ significantly between the three study groups.

Despite being underpowered to detect statistically significant differences in the primary outcome of cesarean delivery rates, several clinically meaningful secondary outcomes emerged from this trial. Combined cervical ripening with a cervical ripening balloon (CRB) for 6 h together with oral misoprostol was associated with a substantially shorter induction-to-delivery interval compared with either method alone, reducing the duration of labor induction by approximately 6–8 h. In addition, the combined approach demonstrated superior early cervical ripening, reflected by higher Bishop scores after 6 h, lower rates of failed induction, and a comparable safety profile across all study groups. These findings further support the effectiveness of CRB placement for only 6 h, rather than the traditionally practiced 12-h duration, as adequate cervical change appears to be achieved within this shorter timeframe. Notably, the Bishop scores achieved following 6 h of CRB placement were similar, and in some cases superior, to those achieved with oral misoprostol alone, consistent with previous studies supporting mechanical cervical ripening methods [5] Although the present study could not reliably determine whether combined cervical ripening reduces cesarean delivery rates, the observed improvements in cervical readiness, induction efficiency, and lower failed induction rates provide biologic and clinical plausibility for this hypothesis. It is possible that more effective and timely cervical ripening may reduce prolonged labor and failed induction, both of which are recognized contributors to intrapartum cesarean delivery. Larger adequately powered trials are needed to determine whether these favorable secondary outcomes ultimately translate into a reduction in cesarean birth rates.

Previous studies have evaluated the combined use of vaginal misoprostol and balloon catheter, compared with vaginal misoprostol alone, with inconsistent results that remain insufficient guide clinical practice [7]. However, evidence regarding the specific combination of 6 h CRB with oral misoprostol is lacking. To the best of our knowledge, this is the first randomized trial to address the effects of this approach.

In recent years, combined methods for cervical ripening have gained increasing attention, and current recommendations from the American College of Obstetricians and Gynecologists support their use [5]. Many patients do not achieve adequate cervical ripening with a single method, and require sequential or crossover intervention, leading to a prolonged induction process. Combining pharmacological and mechanical methods may reduce the need for crossover [13, 14], thereby shortening the induction process and improving efficacy [15, 16].

With the rising prevalence of labor induction, reducing the duration of the induction process has become an important clinical objective. Prolonged inductions are associated with increased resource utilization, greater workload in labor wards, and reduced maternal satisfaction. Therefore, strategies that safely shorten time to delivery while maintaining comparable cesarean delivery rates are of particular clinical relevance.

The overall cesarean delivery rate observed in our study is consistent with previous reports [10, 17], as is time to delivery [18–20], supporting the external validity of our findings. Moreover, our results are aligned with those of a prior randomized controlled trial conducted at our institution [11], further supporting the reproducibility of this approach. The demographic characteristics of our cohort were also comparable to those reported in previous studies [16, 21], although prior trials have predominantly included nulliparous patients, whereas approximately half of our cohort was multiparous.

We also examined the indications for IOL and identified considerable variability across previous studies. However, the distribution of the main indications in our cohort was consistent with previously reported ranges, including postdate (25–60%), hypertensive disorders (10–50%), reduced fetal movements (15%), and glucose abnormalities (6–25%) [16, 22–24]. We additionally report indications such as non-reassuring fetal heart rate, macrosomia, and maternal request, which are less commonly described in the literature.

It is noteworthy that only a minority of studies comparing mechanical and pharmacological induction methods have utilized oral misoprostol, [25, 26] as was done in our study. The oral route may be more acceptable to patients compared with vaginal administration, potentially improving the overall patient experience.

### Strengths and limitations

The main strength of the current study lies in its prospective randomized design, reflecting real life practices and providing evidence on the effectiveness of a 6 h balloon. The similarities of our cohort to previous studies support generalizability. The major limitation of the study is its limited sample size, caused by slow recruitment leading to a premature termination. In addition, some participants (10.4%) have missing primary outcome data, with differential rates across groups. Our complete-case analysis assumes data are Missing Completely At Random (MCAR). However as differential patterns were observed across groups, bias may have contributed to the non-significant primary result. Some data on BMI were also missing.

Recruitment was slower than anticipated for several reasons. Strict inclusion and exclusion criteria limited the eligible population, with only approximately 250 of 1,500 screened women qualifying for randomization, and many otherwise eligible women presented for induction outside the hours when research staff were available to obtain consent and randomize. Enrollment was further constrained by a temporary suspension of recruitment during the COVID-19 pandemic and a months-long shortage of misoprostol. Together, these factors contributed to the premature termination of the trial.

We were unable to blind patients and caregivers to the allocation groups. Results from a single tertiary center may not apply to all institutions. In addition, exclusion of high risk patients such as severe fetal growth restriction and oligohydramnios, may also limit generalizability. A multi-center RCT is required to confirm our findings in diverse populations. Patients satisfaction is also an important outcome and should also be collected in future studies.

## Conclusion

In this randomized controlled trial, the combined treatment did not significantly reduce CD rates. However, this study is likely underpowered to detect clinically meaningful cesarean rate differences. Significant improvements in labor induction efficiency were demonstrated, Combined treatment was associated with a significantly shorter induction-to-delivery interval and improved early cervical ripening. These findings suggest that a combined mechanical and pharmacological approach may enhance the efficiency of labor induction without compromising safety. A larger multi-center RCT is recommended to assess whether combined therapy reduces cesarean delivery rates.

## Supplementary Information

Below is the link to the electronic supplementary material.

Supplementary file1 (DOC 219 KB)

Supplementary file2 (PPTX 36 KB)

Supplementary file3 (DOCX 32 KB)

## Data Availability

Available with restricted access: The Ungdata surveys that support the findings of this study are available from Norwegian Social Research (NOVA), but restrictions apply to the availability of these data, which were used under licence for the current study and so are not publicly available. The data are, however, available upon request and with the permission of Norwegian Social Research (NOVA).
