# Supplementary material for: Labor induction with double balloon catheter, oral misoprostol or both: a randomized controlled trial
Source: Arch Gynecol Obstet. 2026 Aug 12;313(1):308. doi: 10.1007/s00404-026-08526-9 (PMC13748234; doi:10.1007/s00404-026-08526-9)
Supplement: Supplementary file 1 [file 404_2026_8526_MOESM3_ESM.docx]

Supplements

# Supplementary Table S1: Protocol violations

|  | **6hr DB** N = 65*^1^* | **OM** N = 75*^1^* | **DOM** N = 76*^1^* |
| --- | --- | --- | --- |
| **Protocol violation** | 0/65 (0%) | 3/75 (4.0%) | 0/76 (0%) |
| **Spontaneous labor** | 1/65 (1.5%) | 0/75 (0%) | 0/76 (0%) |
| *^1^*n/N (%) | | | |

## Table S2: Demographics and clinical characteristics by parity

|  | **Nullipara** | | | | **Multipara** | | | |
| --- | --- | --- | --- | --- | --- | --- | --- | --- |
| Median (IQR) or n (%) | **6hr DB** N = 37*^1^* | **OM** N = 44*^1^* | **DOM** N = 38*^1^* | **p-value***^2^* | **6hr DB** N = 28*^1^* | **OM** N = 31*^1^* | **DOM** N = 38*^1^* | **p-value***^2^* |
| **Maternal age** | 28 (24, 31) | 30 (27, 34) | 29 (25, 33) | 0.2 | 31 (28, 34) | 31 (28, 36) | 32 (29, 36) | 0.2 |
| **Gestational age (weeks)** | 39.6 (38.2, 40.5) | 39.3 (38.0, 40.3) | 39.6 (38.4, 40.5) | 0.5 | 39.3 (38.5, 40.2) | 39.1 (38.4, 40.0) | 39.4 (38.6, 40.5) | 0.3 |
| **BMI (Kg/m2)** | 29.2 (26.0, 31.6) | 30.9 (27.0, 35.0) | 29.3 (26.5, 33.6) | 0.8 | 30.1 (24.1, 31.3) | 30.0 (27.0, 32.0) | 30.0 (26.0, 35.2) | 0.8 |
| (Missing) | 14 | 11 | 6 |  | 7 | 4 | 7 |  |
| **Bishop score before induction** | 2 (0, 3) | 1 (0, 2) | 2 (0, 3) | 0.8 | 1 (0, 3) | 2 (0, 3) | 2 (0, 3) | 0.6 |
| (Missing) | 0 | 4 | 2 |  | 3 | 1 | 1 |  |
| **Birth weight (g)** | 3,324 (3,033, 3,635) | 3,315 (3,060, 3,680) | 3,358 (3,025, 3,675) | >0.9 | 3,548 (3,178, 3,830) | 3,465 (3,008, 3,665) | 3,493 (3,070, 3,695) | 0.4 |
| (Missing) | 1 | 3 | 0 |  |  |  |  |  |
| **Epidural during labor** | 34 (92%) | 39 (89%) | 37 (97%) | 0.4 | 26 (93%) | 28 (90%) | 34 (89%) | >0.9 |
| **Post date** | 14 (38%) | 10 (23%) | 12 (32%) | 0.3 | 4 (14%) | 6 (19%) | 9 (24%) | 0.6 |
| **Hypertensive disorders** | 6 (16%) | 8 (18%) | 7 (18%) | >0.9 | 1 (3.6%) | 10 (32%) | 1 (2.6%) | <0.001 |
| **Oligohydramnios** | 1 (2.7%) | 4 (9.1%) | 2 (5.3%) | 0.6 | 2 (7.1%) | 0 (0%) | 0 (0%) | 0.081 |
| **Non reassuring fetal heart rate** | 5 (14%) | 3 (6.8%) | 2 (5.3%) | 0.5 | 2 (7.1%) | 2 (6.5%) | 4 (11%) | >0.9 |
| **Macrosomia** | 6 (16%) | 4 (9.1%) | 8 (21%) | 0.3 | 6 (21%) | 5 (16%) | 3 (7.9%) | 0.3 |
| **Reduced fetal Movements** | 3 (8.1%) | 7 (16%) | 4 (11%) | 0.6 | 7 (25%) | 3 (9.7%) | 6 (16%) | 0.3 |
| **Glucose abnormalities** | 6 (16%) | 12 (27%) | 3 (7.9%) | 0.069 | 4 (14%) | 5 (16%) | 10 (26%) | 0.4 |
| **Maternal request** | 0 (0%) | 2 (4.5%) | 3 (7.9%) | 0.3 | 3 (11%) | 4 (13%) | 4 (11%) | >0.9 |
| **Other** | 1 (2.7%) | 3 (6.8%) | 2 (5.3%) | 0.9 | 3 (11%) | 3 (9.7%) | 5 (13%) | >0.9 |
| *^1^*Median (Q1, Q3); n (%) | | | | | | | | |
| *^2^*Kruskal-Wallis rank sum test; Fisher's exact test; Pearson's Chi-squared test | | | | | | | | |

## Table S3: Primary outcome by parity

|  | **Nullipara** | | | | **Multipara** | | | |
| --- | --- | --- | --- | --- | --- | --- | --- | --- |
| n (%) | **6hr DB** N = 37*^1^* | **OM** N = 44*^1^* | **DOM** N = 38*^1^* | **p-value***^2^* | **6hr DB** N = 28*^1^* | **OM** N = 31*^1^* | **DOM** N = 38*^1^* | **p-value***^3^* |
| **Cesarean delivery** | 10 (27%) | 7 (16%) | 14 (37%) | 0.10 | 3 (11%) | 1 (3.2%) | 1 (2.6%) | 0.4 |
| **Indication for cesarean delivery** |  |  |  | 0.7 |  |  |  | 0.4 |
| Non-progressive labor | 4/10 (40%) | 4/7 (57%) | 7/14 (50%) |  |  |  |  |  |
| Non-reassuring fetal heart rate | 6/10 (60%) | 2/7 (29%) | 5/14 (36%) |  | 3/3 (100%) | 1/1 (100%) | 0/1 (0%) |  |
| Other | 0/10 (0%) | 1/7 (14%) | 2/14 (14%) |  | 0/3 (0%) | 0/1 (0%) | 1/1 (100%) |  |
| *^1^*n (%); n/N Non-missing (%) | | | | | | | | |
| *^2^*Pearson's Chi-squared test; Fisher's exact test | | | | | | | | |
| *^3^*Fisher's exact test | | | | | | | | |

## Table S4: Secondary outcomes by parity

|  | **6hr DB** N = 37*^1^* | **OM** N = 44*^1^* | **DOM** N = 38*^1^* | **6hr DB vs. OM** | **6hr DB vs. DOM** | **OM vs. DOM** |
| --- | --- | --- | --- | --- | --- | --- |
| Nullipara | | | | | | |
| **Hours from IOL to delivery** |  |  |  | 0.040 | 0.4 | 0.023 |
| Median (Q1, Q3) | 23.8 (17.7, 29.2) | 28.3 (22.6, 37.9) | 20.9 (13.3, 29.9) |  |  |  |
| (Missing/not applicable) | 4 | 6 | 2 |  |  |  |
| **Hours from IOL to vaginal delivery** |  |  |  | 0.12 | >0.9 | 0.12 |
| Median (Q1, Q3) | 22.5 (17.4, 30.2) | 26.6 (21.9, 37.3) | 21.6 (16.0, 32.4) |  |  |  |
| (Missing/not applicable) | 13 | 12 | 15 |  |  |  |
| **Bishop 2** |  |  |  | <0.001 | 0.3 | <0.001 |
| Median (Q1, Q3) | 6.0 (3.5, 6.0) | 3.0 (2.0, 5.0) | 6.0 (4.0, 7.0) |  |  |  |
| (Missing/not applicable) | 1 | 5 | 3 |  |  |  |
| **Failed induction** | 8 (22%) | 4 (9.1%) | 2 (5.3%) | 0.2 | 0.14 | 0.7 |
| **Oxytocin infusion** | 35 (95%) | 33 (75%) | 35 (92%) | 0.091 | >0.9 | 0.11 |
| **Fetal heart rate decelerations during labor** | 11 (31%) | 12 (30%) | 9 (24%) | >0.9 | >0.9 | >0.9 |
| (Missing) | 2 | 4 | 0 |  |  |  |
| **Tachysystole** | 2 (5.6%) | 3 (7.1%) | 5 (14%) | >0.9 | 0.7 | 0.7 |
| (Missing) | 1 | 2 | 2 |  |  |  |
| **Post-partum hemorrhage** | 2 (5.7%) | 4 (10%) | 5 (13%) | 0.7 | 0.7 | 0.7 |
| (Missing) | 2 | 4 | 0 |  |  |  |
| **Chorioamnionitis** | 4 (11%) | 2 (5.1%) | 5 (13%) | 0.6 | >0.9 | 0.6 |
| (Missing) | 2 | 5 | 0 |  |  |  |
| Multipara | | | | | | |
| **Hours from IOL to delivery** |  |  |  | >0.9 | 0.005 | 0.011 |
| Median (Q1, Q3) | 15.8 (14.2, 20.7) | 17.5 (11.9, 21.3) | 10.9 (9.0, 15.4) |  |  |  |
| (Missing/not applicable) | 5 | 1 | 2 |  |  |  |
| **Hours from IOL to vaginal delivery** |  |  |  | 0.9 | 0.002 | 0.008 |
| Median (Q1, Q3) | 15.8 (14.3, 20.6) | 17.2 (11.9, 21.0) | 10.9 (8.9, 15.0) |  |  |  |
| (Missing/not applicable) | 7 | 2 | 3 |  |  |  |
| **Bishop 2** |  |  |  | >0.9 | 0.017 | 0.017 |
| Median (Q1, Q3) | 5.0 (4.0, 6.0) | 5.0 (4.0, 6.0) | 6.0 (5.0, 7.0) |  |  |  |
| (Missing/not applicable) | 3 | 5 | 4 |  |  |  |
| **Failed induction** | 4 (14%) | 0 (0%) | 0 (0%) | 0.048 | 0.048 |  |
| (Missing) | 0 | 1 | 0 |  |  |  |
| **Oxytocin infusion** | 23 (82%) | 21 (70%) | 22 (58%) | 0.4 | 0.2 | 0.4 |
| (Missing) | 0 | 1 | 0 |  |  |  |
| **Fetal heart rate decelerations during labor** | 7 (27%) | 7 (23%) | 5 (14%) | 0.8 | 0.5 | 0.5 |
| (Missing) | 2 | 0 | 1 |  |  |  |
| **Tachysystole** | 3 (12%) | 4 (13%) | 3 (8.1%) | >0.9 | >0.9 | >0.9 |
| (Missing) | 3 | 1 | 1 |  |  |  |
| **Post-partum hemorrhage** | 2 (8.0%) | 2 (6.7%) | 1 (2.7%) | >0.9 | 0.9 | 0.9 |
| (Missing) | 3 | 1 | 1 |  |  |  |
| **Chorioamnionitis** | 0 (0%) | 0 (0%) | 0 (0%) |  |  |  |
| (Missing) | 3 | 1 | 1 |  |  |  |
| *^1^*n (%) | | | | | | |

## Interaction of parity and treatment

Table S5: Logistic regression model of cesarean section

|  | **OR** | **95% CI** | **p-value** |
| --- | --- | --- | --- |
| **Study group** |  |  |  |
| DB | — | — |  |
| OM | 0.51 | 0.17, 1.50 | 0.2 |
| DOM | 1.58 | 0.59, 4.29 | 0.4 |
| **Parity** |  |  |  |
| Nullipara | — | — |  |
| Multipara | 0.32 | 0.07, 1.20 | 0.11 |
| **Study group * Parity** |  |  |  |
| OM * Multipara | 0.54 | 0.02, 5.97 | 0.6 |
| DOM * Multipara | 0.14 | 0.01, 1.48 | 0.13 |
| Abbreviations: CI = Confidence Interval, OR = Odds Ratio | | | |

The interaction between study group and parity was not statistically significant for cesarean delivery.

Table S6: Linear regression model of time from induction to delivery

|  | **Beta** | **95% CI** | **p-value** |
| --- | --- | --- | --- |
| **Study group** |  |  |  |
| DB | — | — |  |
| OM | 10 | 3.0, 18 | 0.006 |
| DOM | -0.47 | -8.0, 7.0 | >0.9 |
| **Parity** |  |  |  |
| Nullipara | — | — |  |
| Multipara | -7.9 | -16, 0.60 | 0.070 |
| **Study group * Parity** |  |  |  |
| OM * Multipara | -11 | -23, 0.07 | 0.053 |
| DOM * Multipara | -4.2 | -15, 7.0 | 0.5 |
| Abbreviation: CI = Confidence Interval | | | |

The interaction between study group and parity was not statistically significant for induction to delivery time.
